# Large Language Model Architectures in Health Care: Scoping Review of Research Perspectives

**DOI:** 10.2196/70315

**Published:** 2025-06-19

**Authors:** Florian Leiser, Richard Guse, Ali Sunyaev

**Affiliations:** 1 Research Group Critical Information Infrastructures Institute of Applied Informatics and Formal Description Methods Karlsruhe Institute of Technology Karlsruhe Germany; 2 Chair of Information Infrastructures School of Computation, Information and Technology Technical University of Munich, Campus Heilbronn Heilbronn Germany

**Keywords:** large language models, scoping review, ChatGPT, generative artificial intelligence, digital health, medical informatics

## Abstract

**Background:**

Large language models (LLMs) can support health care professionals in their daily work, for example, when writing and filing reports or communicating diagnoses. With the rise of LLMs, current research investigates how LLMs could be applied in medical practice and their benefits for physicians in clinical workflows. However, most studies neglect the importance of selecting suitable LLM architectures.

**Objective:**

In this literature review, we aim to provide insights on the different LLM model architecture families (ie, Bidirectional Encoder Representations from Transformers [BERT]–based or generative pretrained transformer [GPT]–based models) used in previous research. We report on the suitability and benefits of different LLM model architecture families for various research foci.

**Methods:**

To this end, we conduct a scoping review to identify which LLMs are used in health care. Our search included manuscripts from PubMed, arXiv, and medRxiv. We used open and selective coding to assess the 114 identified manuscripts regarding 11 dimensions related to usage and technical facets and the research focus of the manuscripts.

**Results:**

We identified 4 research foci that emerged previously in manuscripts, with LLM performance being the main focus. We found that GPT-based models are used for communicative purposes such as examination preparation or patient interaction. In contrast, BERT-based models are used for medical tasks such as knowledge discovery and model improvements.

**Conclusions:**

Our study suggests that GPT-based models are better suited for communicative purposes such as report generation or patient interaction. BERT-based models seem to be better suited for innovative applications such as classification or knowledge discovery. This could be due to the architectural differences where GPT processes language unidirectionally and BERT bidirectionally, allowing more in-depth understanding of the text. In addition, BERT-based models seem to allow more straightforward extensions of their models for domain-specific tasks that generally lead to better results. In summary, health care professionals should consider the benefits and differences of the LLM architecture families when selecting a suitable model for their intended purpose.

## Introduction

### Background

The last years brought an unmatched rise of large language models (LLMs) such as OpenAI’s ChatGPT [1,2], Google’s Bidirectional Encoder Representations from Transformers (BERT) [3], and Meta’s Llama [4]. LLMs have dramatically extended the abilities of natural language processing through generating text by repeatedly adding the most likely following words [5]. Most LLMs are based on large amounts of general-purpose text data on which the models were trained [6-8]. Thereby, LLMs provide human-like responses to user-given prompts and can analyze [9], explain [10], and generate text [11]. These abilities offer several opportunities to facilitate and improve workflows in various domains, including health care.

To that end, LLMs have been increasingly applied and tested, for example, to assist physicians in diagnosis and treatment decisions [12], researchers in identifying disease phenotypes [13], or students in medical examination preparation [14]. Despite these opportunities, the use of LLMs in health care is subject to biases [15], hallucinations (ie, presenting incorrect information in a factual correct form [14]), or a limited understanding of the complexity of current medical nomenclature [16]. Since health care–specific nomenclature is not fully covered in general-purpose LLMs, their applicability in health care is restricted. In addition, the model architectures of general-purpose LLMs differ. For example, BERT-based models use bidirectional encoding, meaning the input text is read in both directions, making them especially suitable for language understanding [3]. In contrast, generative pretrained transformer (GPT)–based models are built on unidirectional decoders, allowing them to exceed in text generation [1]. If practitioners and researchers in health care use general-purpose LLMs without being aware of their benefits and limits, the LLM’s unsuitability might risk patient health.

With the ever-evolving LLM landscape, it is crucial for health care researchers and practitioners to have a thorough overview and keep up with the current trends of using LLMs in health care. Having insights into models’ performances on specific tasks and the application of LLM architecture families to specific health care improvements could significantly ease the usage of LLMs in health care. However, prior literature reviews focus primarily on issues in applying LLMs in health care and lack a holistic overview of technical instantiations and research foci.

### Prior Work

Due to the underrepresentation of medical nomenclature in the training of most general-purpose LLMs, models used in health care require unique adaptation. One prominent example is Med-PaLM, which can pass questions in the style of the United States Medical Licensing Examination (USMLE) [17]. Medical LLMs have by now been developed and adapted for different use cases such as documentation, communication, decision support, picture interpretation, or treatment plan creation [18].

Previous review studies investigated the opportunities and limitations of LLMs in health care [19-21]. These reviews have started to aggregate the knowledge scattered across various medical specialties and focus on opportunities and risks (eg, hallucinations, legal and ethical concerns, or privacy issues) for using LLMs in biomedicine [21] or on the development and applications of LLMs in health care (eg, information and knowledge discovery) [20]. These reviews have already provided a considerable description of the applications of LLMs and related them to the patient care journey [20,21]. For specific applications, one review also included an overview of selected LLM architectures in biomedical and health fields alongside their performance [21]. These reviews made a critical step in providing an overview of the emerging application areas and the performance of LLM architectures for specific biomedical datasets [20,21]. However, they focus on a selection of LLMs and do not include the breadth of available LLM architectures in health care research. Moreover, the application areas to which the LLM architectures are applied could help researchers and practitioners to choose the right LLM architecture for a specific use case. Since LLM architectures differ in how they process text [1,3], specific architectures can be more suitable for a particular medical task. Hence, a thorough investigation of current LLM architectures in health care is crucial as it can aid practitioners in selecting suitable LLM model architectures for their medical tasks.

### Objectives

In this study, we investigate the use of LLM model architectures in current studies in health care. While our results regarding the target use are similar to other reviews [21], we specifically provide an analysis of model architecture families for 4 research foci (ie, LLM Performance, LLM Societal Impact, LLM Comprehensibility, and LLM Innovation). For each research focus, we assess the used model architectures, the integration of LLMs in clinical practice, and the data types used in each manuscript. With that, we aim to provide insights into LLMs’ maturity, practical implementation, and innovation stages in health care. We conduct a scoping review [22] to determine the breadth and boundaries of the literature on model architectures and the use of LLMs in the medical domain [23].

## Methods

In our study, we investigated PubMed as the primary database for identifying manuscripts on LLMs in health care. Since the topic is emerging and novel models are introduced rapidly, we also included the preprint databases arXiv and medRxiv in our search. We searched for *TITLE-ABS-KEY(GPT OR LLM? OR Large Language Model? OR LLaMA OR Bard OR Med-PaLM)* to focus on LLMs rather than the medical domain. We included specific model architectures in our search string to gain insights into the technical manifestations of widely recognized LLMs. Our initial search in January 2024 yielded 1842 hits, with arXiv having the most results (n=813). Before screening the manuscripts, we excluded 479 manuscripts published before 2018, which marked the introduction of GPT-1 [24] as well as duplicates (n=117) and non-English publications (n=37).

For analyzing the manuscripts, we followed the PRISMA-ScR (Preferred Reporting Items for Systematic Reviews and Meta-Analyses extension for Scoping Reviews) guidelines that are summarized in Multimedia Appendix 1 [25]. During the screening of the title and the abstract of each manuscript, we investigated the topic of the manuscripts and excluded papers with no focus on health care (n=837). We aligned the decisions on borderline cases by having 2 authors (FL and RG) investigate the manuscript and reach a consensus. Excluded manuscripts were, for example, related to Llamas instead of the Llama architecture.

In addition, we excluded papers with no focus on LLMs (n=206) or gray literature (n=14). We also found 4 additional duplicates. During full-text analysis of the remaining 148 manuscripts, we further found 15 manuscripts with no focus on health care, 2 manuscripts with no LLM focus, 16 gray literature manuscripts such as blog posts, and excluded 1 publication with access restrictions. All included manuscripts can be seen in Multimedia Appendix 2.

Our scoping review used a combination of predefined categories and open coding as shown in Table 1. We coded the identified manuscripts along 9 dimensions split between 3 usage, 5 technical dimensions, and an overarching *research focus* dimension. To align the understanding of all dimensions and generate codes inductively, we double-coded 20 manuscripts during full-text analysis (17.5%, 20/114). For these 20 manuscripts, we ensured that the author team understood the dimensions and their codes coherently. With the initial set of dimensions and codes, we proceeded with single coding for all dimensions. The coding process was monitored through regular meetings where we discussed coding of all dimensions. If the coding author was uncertain about a dimension, other members of the author team reinvestigated the manuscript and decided. We introduced new codes only if the entire author team agreed. In coding the identified manuscripts, we allowed multiple codes within each dimension to represent the variety of characteristics discussed within the manuscripts. Therefore, most coding dimensions excel 114, for example, since studies use multiple data modalities or LLM architectures.

**Table 1:** Overview of coding dimensions for the scoping review.

Coding dimension			Description
**Usage**			
	Medical specialty	Medical domain of the conducted study	
	Target audience	Stakeholders targeted by the LLM^a^ application	
	Target use	Use case of the LLM application	
**Technical**			
	Model integration	Focus of the LLM application	
	Model novelty	Maturity of model development	
	Model architecture	Applied LLM architecture	
	Data type	Used data type	
	Evaluation metrics	Used evaluation metrics	
	Research theme	Investigated improvement focus of the manuscript	

^a^LLM: large language model.

An overview of all dimensions and corresponding tables and figures can be found in Multimedia Appendix 3. For usage dimensions, we investigated *medical specialty* (eg, radiology, pathology, or cardiology), *target audience* (eg, physicians, patients, or students), and *target use* (eg, diagnostic support or patient communication). These dimensions provided relevant information for the targeted application of LLMs in health care.

We further investigated the technical aspects of each manuscript. The coding dimension *model integration* differentiated between studies focusing on LLMs on a conceptual level, their usage, implementation, or evaluation. Relatedly, we captured the *model novelty* (ie, developing a new model, applying an existing model, extending an existing model, or not using a model at all). This categorization helped us understand the extent of current and new developments of LLMs in health care. Furthermore, we extracted the specific *model architectures* such as GPT-3.5 or BERT to highlight the variety of models in the manuscripts. We also noted the *data type* used in the studies such as clinical notes, images, or electronic health records (EHRs). We documented the *evaluation metrics* used to assess the models’ performance, such as accuracy, precision, recall, or *F*_1_-score.

Finally, we coded the targeted contributions of the manuscript as research themes. Similar to the other coding dimensions, we openly coded the research themes (eg, correctness and patient well-being). Since several contributions discussed across the identified research themes were within similar contexts (ie, fairness: n=1, bias: n=4, and societal impact: n=2), we combined and aggregated coding themes into research foci. After revising the research themes and double-coding by the involved authors, we combined the themes into 4 research foci.

## Results

**Figure 1:**
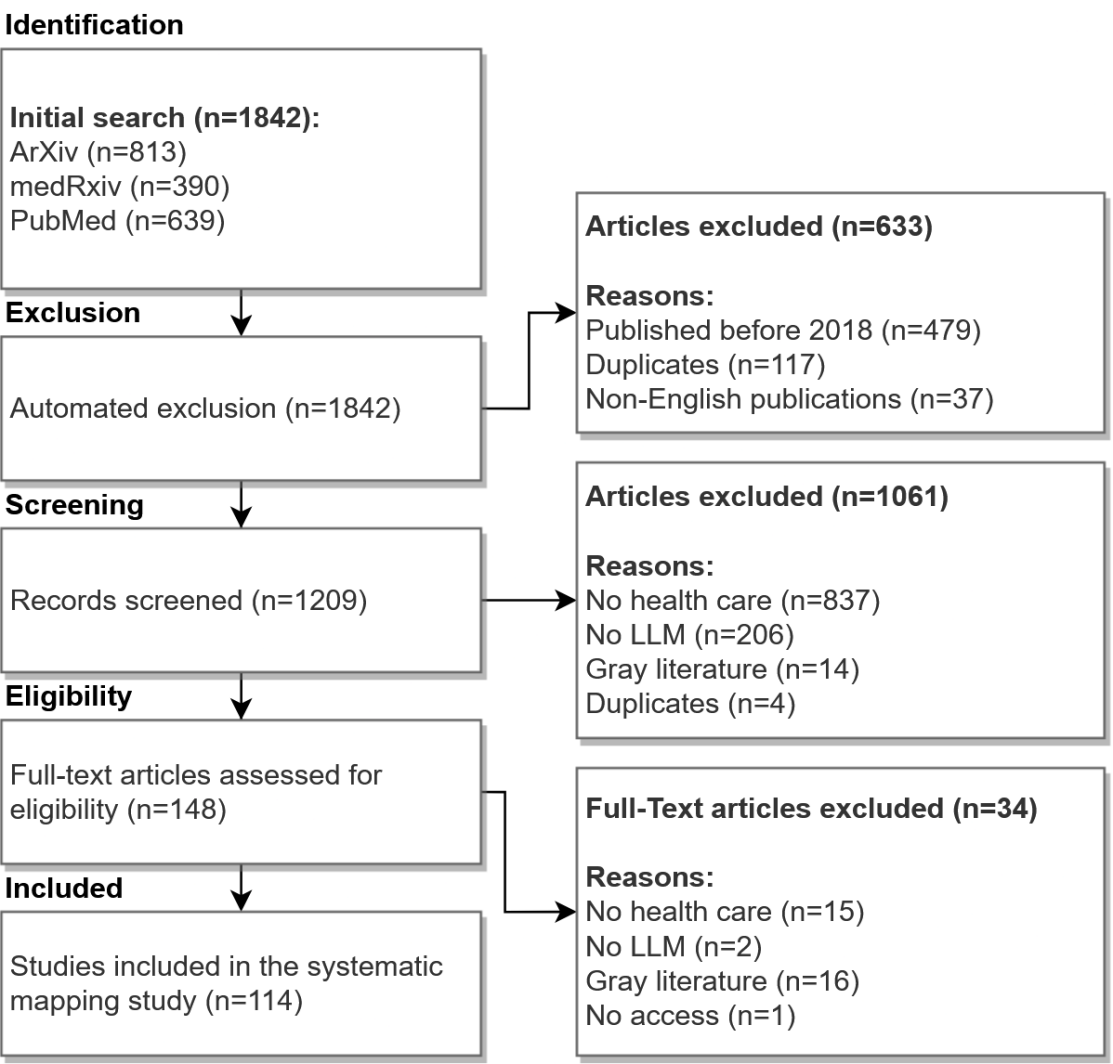
PRISMA-ScR (Preferred Reporting Items for Systematic Reviews and Meta-Analyses extension for Scoping Reviews) flowchart for inclusion and exclusion of manuscripts. LLM: large language model.

Our scoping review was carried out with the PRISMA-ScR guidelines (see Figure 1 for the PRISMA-ScR flowchart).

### Usage Dimensions

#### Medical Specialty

In examining the medical specialty (Table 2), we find a frequent usage of LLMs in medical research (26/114, 22.81%), health education (17/114, 14.91%), and hospital medicine (17/114, 14.91%). LLMs in medical research are used for predictions [26-29], labeling [27,30,31], or fine-tuning techniques [32,33] without focusing on a specific medical domain. Most studies using LLMs in health education (15/17, 88.23%) examine the performance of LLMs in medical domain-agnostic examinations such as the USMLE [34], the Medical College Admission Test (MCAT) [35], or the Chinese National Medical Licensing Examinations [36]. Other studies investigating LLMs for medical examinations conduct domain-specific examinations such as neurology board questions [37]. Studies that focus on hospital medicine investigate the usage of LLMs as a replacement for doctors’ medical advice [33,38-40].

**Table 2:** Medical specialty.

Medical specialty	Values, n (%)
Anesthesiology	3 (2.63)
Cardiology	6 (5.26)
Clinical laboratory sciences	6 (5.26)
Dermatology	3 (2.63)
Emergency medicine	2 (1.75)
Endocrinology	3 (2.63)
Gastroenterology	4 (3.51)
Health education	17 (14.91)
Hospital medicine	17 (14.91)
Intensive care medicine	2 (1.75)
Internal medicine	7 (6.14)
Medical research	26 (22.81)
Nephrology	2 (1.75)
Neurology	6 (5.26)
Neurosurgery	3 (2.63)
Oncology	6 (5.26)
Ophthalmology	5 (4.39)
Orthopedic surgery	1 (0.88)
Otolaryngology	2 (1.75)
Pathology	5 (4.39)
Pediatrics	2 (1.75)
Psychiatry	4 (3.51)
Pulmonology	2 (1.75)
Radiology	7 (6.14)

#### Target Use

In our sample, we identify 9 target uses of applying LLMs in health care. Within our samples of 114 studies, we identify 161 instances of LLM target use cases in total. The most prominent target use was decision support in 27.19% (31/114) of the studies, followed by information retrieval (24/114, 21.05%) and communication (24/114, 21.05%). Target use is related to our research foci, where the application of LLMs for communication and explanation targets at LLMs comprehensiveness. For example, Lim et al [41] compare the perceived accuracy and comprehensiveness of GPT-3.5, GPT-4, and BARD on myopia-related questions.

#### Target Audience

We identify 8 different stakeholders in our sample. Most studies provide insights for physicians (69/114, 60.53%), researchers (32/114, 28.07%), patients (23/114, 20.18%), and students (17/114, 14.91%). Concerning the research foci, we find that physicians are more targeted in studies on the comprehensibility of LLMs (13/16, 81.25%), for example, to understand breast cancer survival [42]. Students are especially targeted for educative purposes to assess passing current medical licensing examinations with LLM support [34,43].

### Technical Dimensions

#### Model Integration

In our sample, 2 studies (2/114, 1.75%) focus conceptually on LLM usage from an ethical or regulatory viewpoint [15,44]. Most manuscripts focus on using LLMs in clinical practice (65/114, 57.02%). Twenty-seven (23.68%) studies implement LLM models by extending existing LLM architectures or developing new models. However, only 2 manuscripts report on a full implementation already evaluated in a real-world setting [29,45]. Twenty papers (20/114, 17.54%) evaluate different model architectures, for example, to investigate racial presumptions in Claude, BERT, GPT-3.5, and GPT-4 [46].

#### Model Novelty

Most papers apply LLMs (86/114, 75.44%) without focusing on technical changes, and only a fourth of the manuscripts extended existing models (29/114, 25.44%) by incorporating additional data, knowledge, or human assessment with 6 manuscripts doing both. We identify only 3 (2.63%) studies developing novel retrained model architectures in our sample. The 3 models include chain-of-thought-reasoning into the inference step of LLMs [47], incorporate physician behavior to teach ChatGPT to reply in a doctor-specific manner [38], or retrain GPT-3 on biomedical datasets containing clinical texts by question-answer challenges [48].

Most model extensions are part of the LLM innovation research focus. While most studies use GPT-based architectures, studies in the innovation research name 64 model architectures, using on BERT-based and GPT-based models 23 times (23/64, 35.94%) each. The higher relative percentage indicates that BERT-based models are easier to extend for novel models than GPT-based models.

#### Model Architecture

We observe that authors frequently apply multiple LLM architectures. This resulted in 57 different LLM architectures used across all studies. Thirty-four of these architectures are named only once, highlighting the variety of LLMs developed for health care. In general, GPT-based (139/259, 53.66%) and BERT-based models (66/259, 25.46%) provide the vast majority of applied LLM architectures. Most of the studies apply OpenAI’s state-of-the-art models GPT-3 (n=12), GPT-3.5 (n=74), and GPT-4 (n=41), although BERT (n=17) and Llama (n=10) are frequently used as well. An overview of the different model architecture families and their use in different research foci is shown in Table 3.

**Table 3:** Number of manuscripts per large language model architecture families and research focus.

Large language model (LLM) architecture family	LLM performance	LLM societal impact	LLM Comprehensibility	LLM innovation
BARD	6 (2.67)	1 (2.13)	1 (3.33)	2 (3.13)
BERT	62 (27.56)	8 (17.02)	6 (20.00)	23 (35.94)
GLM	3 (1.33)	0 (0.00)	0 (0.00)	2 (3.13)
GPT	119 (52.89)	31 (65.96)	17 (56.67)	23 (35.94)
LaMDa	2 (0.89)	0 (0.00)	0 (0.00)	0 (0.00)
Llama	9 (4.00)	2 (4.26)	4 (13.33)	5 (7.81)
T5	5 (2.22)	1 (2.13)	0 (0.00)	0 (0.00)
Vicuna	6 (2.67)	0 (0.00)	0 (0.00)	4 (6.25)
Other	13 (5.78)	4 (8.51)	2 (6.67)	5 (7.81)

#### Data Types

Our sample accounts for multiple data modalities used as input or output dimensions of the LLMs. This results in 200 mentions of 11 data modalities within our sample as shown in Table 4. The most significant data type manifests as symptom descriptions (n=44), treatment options (n=31), and diagnosis (n=29). Since these modalities comprise the steps during diagnosis, they are also frequently used in combination, for example, to assess the treatment advice generated by ChatGPT if patients present themselves with symptom descriptions [40]. These data are also heavily used in manuscripts investigating the understandability of LLMs. The understandability of LLM is especially important when the models communicate their reasoning with patients. Interaction with patients, in turn, is required when treatment options, diagnosis, or symptom descriptions are provided.

**Table 4:** Data types used in the retrieved literature.

Data type	Values, n (%)
Adverse effects	7 (6.14)
Clinical notes	12 (10.53)
Diagnosis	29 (25.44)
EHR^a^	13 (11.4)
Examination questions	28 (24.56)
Genomic data	2 (1.75)
Images	6 (5.26)
Patient communication	7 (6.14)
Reports	21 (18.42)
Symptom description	44 (38.6)
Treatment options	31 (27.19)

^a^EHR: electronic health record.

In contrast, only very few manuscripts focus on traditional medical data modalities such as genomic data (n=2), imaging (n=6), or EHRs (n=13). These data modalities are usually used by physicians and, therefore, are used in innovation-driven papers, such as those by Gao et al [32], who extend GPT-3.5 based on existing patient-doctor dialogues to generate a response for the diagnosis report given an ophthalmology image. The remaining papers revolve around traditionally text-related data such as patient communication or questions (n=8), clinical notes (n=12), or medical reports (n=21). Other frequently used data modalities include examination questions (n=28), for example, the performance of GPT-3.5 in the USMLE [34], the MCAT [35], or on Korean general surgery board examinations [49].

### Research Foci

In our analysis, we analyzed the targeted improvements of the manuscripts. We found 12 research themes that we aggregated into 4 research foci as shown in Table 5. Half of the 114 included manuscripts encompass multiple research foci (n=57).

**Table 5:** Overview of identified research foci and their themes.

Focus and theme			Goal		Values, n (%)
**LLM^a^ performance**					
	Correctness	Ensuring predictive performance		89 (78.07)	
	Performance improvement	Improving LLM performance		14 (12.28)	
	Hallucinations	Analyzing and preventing hallucinations		9 (7.89)	
	Consistency	Comparing LLM performance across contexts or over years		8 (7.02)	
**LLM societal impact**					
	Ethics	Discuss ethical questions for LLM use in high-risk and sensitive contexts of health care		12 (10.53)	
	Bias	Evaluate and mitigate biases such as stereotypes influencing health-related examinations in LLM		6 (5.26)	
	Confidentiality	Evaluate risks and attack vectors in LLM use for health care data and discuss potential solutions		4 (3.51)	
	Regulation	Discuss issues related to accountability and transparency requirements that need to be enforced by law to safely use LLMs in health care		3 (2.63)	
**LLM comprehensibility**					
	Explainability	Enhance the plausibility and comprehensibility of health information provided by LLMs with additional explanations		12 (10.53)	
	Interpretability	Retrace the inner mechanics of the reasoning processes of the LLM		5 (4.39)	
**LLM innovations**					
	Novel applications	Develop novel LLM applications in various health care use cases and research		14 (12.28)	
	Novel training techniques	Improve and develop novel training techniques for LLMs, for example, to improve the quality of LLM responses		10 (8.77)	

^a^LLM: large language model.

#### Research Focus 1: LLM Performance

The research focus on LLM performance encompasses improvements in model performance, ensuring correctness (including hallucinations) and assessing prediction consistency. It underscores the importance of building models that improve performance by providing correct, hallucination-free, and consistent results. Overall, the studies show that domain-specific models improve performance and correctness.

#### Correctness

Ensuring correct predictions of LLMs constitutes the most prominent research theme. Correct predictions are especially crucial in health care as errors can lead to severe consequences for patients such as inappropriate treatment. Eighty-nine manuscripts [50-52] test the correctness of LLMs. However, the predictive performance of LLMs varies based on the source and structure of the input data [53].

The potential of general-purpose LLMs for medical applications is highlighted in a manuscript that explores whether ChatGPT could generate helpful suggestions for improving a clinical decision support system. Clinicians rated the generated suggestions as highly relevant and clear, with low bias [54]. Other manuscripts evaluate the performance of general-purpose LLMs on medical and nursing examinations, for example, on the faculty of public health’s diplomat examination [55]. However, for health care–specific tasks, such as biological named entity recognition [56], general-purpose LLMs perform insufficiently.

Therefore, another common theme is the development of specific-purpose LLMs and testing their correctness. For example, HuatuoGPT extends ChatGPT with real-world data from doctors to outperform other LLMs in terms of human evaluations and benchmarks, yielding highly accurate results in medical contexts [38]. Another study tests the application of LLMs in clinical trial enrollment. For that, GPT-3.5 is used to extract medical entity tags from patient information and classify these to identify suitable clinical trials. The application achieves a high accuracy for this task [57].

#### Performance Improvement

Within the 14 studies in our sample, performance improvement is achieved by adapting LLMs to a specific domain or developing new, specialized LLMs with domain-specific data in LLM pretraining. GatorTronGPT is one example of a clinical LLM trained on clinical and general English text using a GPT-3 architecture and outperforms general-purpose LLMs in medical research [48]. Furthermore, domain adaptation of LLMs improves the predictive performance, for example, to classify nuclear medicine reports [58]. One manuscript compares models ranging from 220 million to 175 billion parameters and finds that smaller, domain-specific models often match or exceed the performance of larger general-purpose models [50].

#### Hallucinations

In our sample, we found 9 studies investigating hallucinations of LLMs. Hallucinations occur especially in summaries of textual information [59] or in 12.5% of GPT-based cancer treatment recommendations [60]. Another study finds that using LLMs for drug-drug interactions also produced many hallucinations [61].

#### Consistency

The consistency of LLMs evaluates performance across different contexts or over time. One manuscript evaluates ChatGPT’s performance on the Japanese National Nursing Examination across multiple years [62]. In another case, researchers assess the consistency across GPT-3.5 and GPT-4 on questions related to heart failure [63]. Overall, the importance of reproducibility in complex decision-making tasks is highlighted [44].

#### Research Focus 2: LLM Societal Impact

The second research focus pertains to the societal impact of LLMs. The societal considerations surrounding LLMs in health care highlight complex challenges related to ethics, bias, confidentiality, and regulation. While LLMs have the potential to improve health care provision, their deployment must be carefully managed to mitigate bias, ensure patient data confidentiality, and ensure ethical appropriateness. Moreover, the absence of clear regulations and accountability frameworks presents a critical barrier to their widespread and safe use.

#### Ethics

Twelve studies investigate ethical concerns such as appropriate empathy of LLMs. For instance, 1 manuscript finds that LLM responses to patient questions express appropriate empathy [39]. On the contrary, another manuscript suggests that LLMs fail to resolve high-risk scenarios, such as suicide prevention [64]. In their manuscript, only a few models provide essential crisis resources such as suicide hotlines, highlighting the need for model refinement to comply with ethical standards [64].

#### Bias

Six studies show that LLMs can replicate and amplify harmful stereotypes in medical content. For example, LLMs propagate race-based misconceptions when responding to historically involved race-based medicine [46]. Another study shows location-based biases in cardiovascular disease risk assessment by ChatGPT [65]. Bias extends beyond location, for example, to gender as ChatGPT changes its predictions more frequently when race or gender descriptors are added to medical texts [66]. While these findings highlight the importance of addressing bias in LLMs to prevent exacerbating health care inequalities, research also discusses potential solutions. For example, LLMs are shown to help identify bias within a corpus of text [30].

#### Confidentiality

Ensuring data confidentiality is still an issue for LLMs in health care [56]. Studies highlight how adversarial attacks could be used to generate misinformation or leak private patient data [67]. Research tries to tackle these issues with a privacy-aware data augmentation approach for LLM-based patient-trial matching [68] or using GPT-3.5 to identify confidential content within clinical notes [69].

#### Regulation

Three studies emphasize the need for better regulation to ensure safe usage of LLMs in health care [15,59,70]. This lack of regulation poses questions about who is held accountable for errors in diagnosis or treatment recommendations [15,59] and the transparency of LLM functionality [70].

#### Research Focus 3: LLM Comprehensibility

The third research focus deals with the comprehensibility of LLMs, particularly in terms of explainability and interpretability. On the one hand, explainability provides insights into LLM predictions and recommendations. Interpretability, on the other hand, offers explanations for the internal processes of LLMs. Both explainability and interpretability are crucial for health care, as health care professionals need to rely on clear, accurate information to make decisions.

#### Explainability

In our sample, 12 studies focus on improving the explainability of LLMs either by enhancing LLMs with additional information or using self-explanations. For example, LLMs incorporate additional information in the form of in-text citations and references [70] or include causal graphs to explore the effect of different genetic perturbations on the survival rates of patients with breast cancer [42].

LLMs self-explaining their reasoning helps students understand complex medical conditions and generate personalized explanations of MCAT-related questions [35]. Similarly, 1 manuscript focuses on using LLMs to explain its reasoning for diagnosing certain mental health conditions [71].

#### Interpretability

Five studies focus on the inner reasoning processes of LLMs. For example, 1 manuscript assesses the reasoning of ChatGPT behind the responses for 119 public health examination questions [55]. In another study, model interpretability is enhanced by using the Alpaca architecture to map radiologist findings to radiological images easing comprehensibility for physicians [12]. Another approach uses GPT-3 to generate interpretability by grouping patients based on the similarity of their embeddings for medication prescriptions [72].

#### Research Focus 4: LLM Innovation

The fourth research focuses on LLM innovation through novel applications and training techniques. This focus explores the adaptation and extension of LLMs to accelerate, improve, and automate health care tasks. At the same time, novel training techniques, such as multimodal learning and context-enriched approaches, are extending the capabilities of LLMs to more complex medical contexts.

#### Novel Applications

Fourteen studies present novel applications of LLMs in specific areas. One manuscript reports that LLMs could reduce the dimensionality of complex health care data such as *ICD-10* (*International Statistical Classification of Diseases, Tenth Revision*) codes while maintaining the original embeddings [73]. Another application uses LLMs to create patient clusters based on pharmacy records for more personalized treatment recommendations [72]. In biomedical research, LLMs trained on additional medical literature identify and validate disease-relevant targets [26]. Studies also show that LLMs can be used for theme-driven analyses (eg, of opioid use disorder) uncovering both frequent and clinically undocumented patterns [27].

In health care education, LLMs are used to create examination questions to accelerate examination generation, standardize assessments, and reduce bias in questions [43]. When preparing for examinations, LLMs could respond to individual learning styles and needs in various medical domains [39,74].

#### Novel Training Techniques

Ten studies in our sample develop novel training techniques to adapt LLMs to health care. Some of these approaches [32,50,58] show that medical data can be directly used to pretrain LLMs. Adapting LLMs or prompts to medical domains improves their performance [32,47,75,76] and could simulate expert thought processes [47].

In a more general setting, the research explores the integration of external knowledge sources to improve the accuracy of Llama in diagnosing disease symptoms [33] or the reliability of ChatGPT responses [70]. Furthermore, LLMs are used to clean and annotate medical data to fine-tune additional LLMs [75].

Other studies develop a novel multimodal model fine-tuned on images, diagnostic reports, and LLM-based dialogues to enhance condition diagnosis and response generation [32,76]. These multimodal training approaches provide LLMs with a broader range of inputs to increase diagnosing versatility.

## Discussion

### Principal Results

With the rise of LLMs in the past years, research using LLMs in health care has significantly increased. We found that the manuscripts in our literature sample targeted a broad variety of stakeholders in the health care sector such as physicians (69/114, 60.53%), researchers (32/114, 28.07%), patients (23/114, 20.18%), or medical students (17/114, 14.91%).

We identified 4 primary research foci for LLMs in health care: LLM performance, LLM societal impact, LLM comprehensibility, and LLM innovation. With several manuscripts tackling 2 research foci simultaneously, most of our manuscripts focused on the performance of LLMs (87/114, 85.09%).

A large number of studies focusing on LLM performance show the importance of accurate, reliable LLM results in paving the way for the application of LLMs in health care. This research focus supports the idea that general-purpose LLMs are not yet ready to be applied to health care tasks and instead need domain adaptation. As the performance of general-purpose LLMs progresses, we found that the predictive performance seems to be better in BERT-based models when investigating a limited sample of 13 manuscripts that used BERT-based and GPT-based models. While the performance difference was often marginal, GPT-based models seem to have a higher recall but lower precision [13,27] or are used to augment training data for BERT-based models [75,77]. This could result from the model architectures. While both model architectures use transformer techniques, BERT-based models use a bidirectional encoder [3] and GPT-based models use a unidirectional decoder [1]. Therefore, BERT can consider words in both reading directions when understanding text, while GPT processes text unidirectionally (usually from left to right) and is optimized for generating texts. This shows an interesting conflict of LLM architectures and highlights the benefits of BERT-based models for medical tasks such as knowledge discovery or report generation [75], while GPT-based models are better suited for language generation and communicative purposes (eg, examination preparation [14] or dialogues with patients [78]).

In addition, the consistency of LLMs was rarely investigated in our sample. Systematic evaluation over time is missing to allow reliable decisions on the application of LLMs. Some studies have started to compare domain-adapted models against domain-agnostic models [56,79]. Our findings suggest that domain-adapted BERT-based models provide the best predictive performance. Further research should strengthen these findings by conducting computational benchmark evaluations between models. Relatedly, hallucinations were evaluated only for general-purpose LLMs in the retrieved manuscripts. Moreover, many hallucinations were present in the target use case of text summarization, although the textual information was directly given to the LLM. For other use cases (eg, knowledge discovery), hallucinations have not been examined in our sample. Several mitigation strategies for hallucinations were developed, such as enabling users to detect hallucinations themselves [80] or reinforcing model inference by incorporating human feedback [80]. As a second research focus, the societal impact of LLMs was investigated (25/114, 21.93%). Our results hint that LLMs in health care come along with race-based misconceptions that reproduce harmful stereotypes [46] and provide insufficient support in high-risk scenarios such as suicide prevention [64]. Similar to research on artificial intelligence, societal and ethical impact should be considered when using LLMs in health care [81]. This indicates that further research is needed to determine how bias in health care can be prevented. This is in line with existing reviews [19-21] that also note various challenges of applying LLMs in health care (eg, hallucination, fairness, bias, privacy, and legal concerns) underlying the relevance of this research focus. In our sample, societal impact is predominantly evaluated with GPT-based models instead of other model architectures such as BERT. When accounting for the societal impact of LLMs, an investigation of multiple model architectures could provide new insights. Further manuscripts highlighted confidentiality and privacy issues when using and adapting LLM with sensitive health care data. Our results indicate that future research still needs to determine privacy-preserving strategies when using LLMs in health care. A possible avenue for future research could be to introduce privacy-preserving training approaches such as federated learning that have been shown to work in health care settings [82]. The third research focus on model comprehensibility included a limited number of 16 studies (16/114, 14.04%). In this selection, a minimal tendency can be made toward extending state-of-the-art LLM models instead of simply applying them. In contrast, despite this limited number of manuscripts, the focus suggests that LLM comprehensibility is tailored toward physicians in 13 of 16 (81.25%) studies. In addition, the frequent use of LLMs for decision support (9/16, 56.25%) and classification tasks (4/16, 25.00%) further emphasizes explanations targeted toward medical personnel. While artificial intelligence research has already highlighted the need for explanations on model reasoning to improve model reliance [83], the explanations should not only adhere the physician’s perspective but also include other stakeholders. Patients’ perspectives should mainly be investigated when assessing model comprehensibility. To that end, our sample suggests that the comprehensibility studies instead comprised patient-specific data types such as symptom descriptions (8/16, 50.00%), treatment options (7/16, 43.75%), EHR (3/16, 18.75%), or images (3/16, 18.75%). As LLMs become increasingly integrated into health care, improving explainability and regulations will be crucial to ensure the safe and responsible use of LLMs and create an environment where LLMs can be used effectively in health care settings [78].

As a fourth research focus, we identified studies focusing on model innovation. In our literature review, we found that 75.44% of the studies applied existing models and 25.44% of the studies extended models (with 6 studies doing both). The studies in our sample rarely implemented new models (2.63%). The studies mostly used state-of-the-art general-purpose models such as GPT-3.5, GPT-4, or BERT. While this holds true for the entire dataset, innovation (ie, model innovation or novel applications) was comparatively more performed on BERT-based models (35.94%) than on GPT-based models (35.94%). This includes health care–specific extensions of LLMs such as BioBERT, ClinicalBERT, or MedBERT that enhance the performance of these general-purpose models for the health care domain by including external knowledge sources. This again builds on the aforementioned differences in model architectures where BERT-based model architectures are better suited for knowledge discovery tasks and GPT-based model architectures rather than solving communicative purposes.

These insights help practitioners to select appropriate LLM model architectures. If the focus of the usage of the LLM is to ease communication, GPT-based models seem more appropriate. When LLMs are used for innovative purposes (such as knowledge discovery), our sample suggests the superiority of BERT-based models. However, most general-purpose models still require domain adaptation and could benefit from the incorporation of domain knowledge [45,84].

### Limitations

This study is not without limitations. First, all codings and assessments of the dimensions were subjectively assessed by the authors of this manuscript. While we tried to minimize the subjectiveness through double-coding and regular meetings to discuss and ensure a similar understanding of all dimensions and manifestations, the final assessment is still subject to subjective assessments. Second, our comparison of GPT-based and BERT-based models focuses on a limited sample of 13 manuscripts that report on the performance of both. Future benchmark evaluations of these manuscripts are needed to support these tendencies. Third, we conducted the assessment based on a literature search in January 2024. While we included early works from prepublication databases such as arXiv or medRxiv to reduce the impact of later publication, maintaining a current and up-to-date review in this fast-changing field is challenging. For example, the introduction of GPT Playground might have facilitated the implementation and adaptation of GPT-based models. Fourth, in our search term, we focused on LLMs and did not include further related concepts (eg, transformer-based models). Although we included established LLMs in our search query, this might have led to the exclusion of relevant studies. Finally, we assume that manuscripts retrieved from prepublication databases have sufficient scientific rigor. While we excluded only 2 publications that we deemed of insufficient quality, manuscripts included in our sample might not hold up to highest scientific standards.

### Conclusions

In this literature review, we identified 4 research foci for LLMs in health care and highlighted specifics to each focus. Most manuscripts in our review focused on the performance of LLMs. Our sample shows a tendency that GPT-based models provide higher recall and are more heavily involved in societal questions that require interaction and communication. In contrast, BERT-based models show higher precision and are more intensely used for innovation such as classification or knowledge discovery. This is in line with the architectural design of the models where the unidirectional design of GPT is better suited for generative purposes. In contrast, the bidirectional design of BERT is better suited for question-answering. We also find that more manuscripts use diagnosis-related data such as treatment recommendations or symptom descriptions than manuscripts using traditional medical data such as images or EHR. This, again highlights the novel ways to communicate with LLMs. While these findings seem to hold through the manuscripts comparing different LLMs, future research should conduct thorough comparative benchmark evaluations.

With this review, we aid health care professionals in understanding the benefits of specific LLM technologies. GPT-based models are better suited for communicative purposes due to their enhanced ability to generate text. However, in the current state, general-purpose LLMs might be able to pass medical examinations but are unable to explain or reason their predictions. When incorporating medical-specific knowledge, the performance of LLMs was further increased. Since we investigate only published literature, future research should extend the comparison of performance and bias of domain-adapted and general-purpose LLM models. Without these benchmarks, we should teach physicians to use LLMs as supporting tools while raising awareness to potential model insufficiencies.

## Appendix

Multimedia Appendix 1 PRISMA-ScR (Preferred Reporting Items for Systematic Reviews and Meta-Analyses extension for Scoping Reviews) checklist.

Multimedia Appendix 2 Overview of all included publications along with our coding dimensions and our analysis of each dimension for each research focus.

Multimedia Appendix 3 Additional tables and figures for the analysis of each dimension.

## Abbreviations

BERT: Bidirectional Encoder Representations from Transformer
EHR: electronic health record
GPT: generative pretrained transformer
ICD-10: International Statistical Classification of Diseases, Tenth Revision
LLM: large language model
MCAT: Medical College Admission Test
PRISMA-ScR: Preferred Reporting Items for Systematic Reviews and Meta-Analyses extension for Scoping Reviews
USMLE: United States Medical Licensing Examination
