# Supplementary material for: Large Language Model Architectures in Health Care: Scoping Review of Research Perspectives
Source: J Med Internet Res. 2025 Jun 19;27:e70315. doi: 10.2196/70315 (PMC12226782; doi:10.2196/70315)
Supplement: Multimedia Appendix 3 [file jmir_v27i1e70315_app3.docx]

## Usage Dimensions

### Medical Specialty

Table S1. Medical specialty.

| **Medical Specialty** | **n (in %)** |
| --- | --- |
|  |  |
| Anesthesiology | 3 (2.63) |
| Cardiology | 6 (5.26) |
| Clinical Laboratory Sciences | 6 (5.26) |
| Dermatology | 3 (2.63) |
| Emergency Medicine | 2 (1.75) |
| Endocrinology | 3 (2.63) |
| Gastroenterology | 4 (3.51) |
| Health Education | 17 (14.91) |
| Hospital Medicine | 17 (14.91) |
| Intensive Care Medicine | 2 (1.75) |
| Internal Medicine | 7 (6.14) |
| Medical Research | 26 (22.81) |
| Nephrology | 2 (1.75) |
| Neurology | 6 (5.26) |
| Neurosurgery | 3 (2.63) |
| Oncology | 6 (5.26) |
| Ophthalmology | 5 (4.39) |
| Orthopedic Surgery | 1 (0.88) |
| Otolaryngology | 2 (1.75) |
| Pathology | 5 (4.39) |
| Pediatrics | 2 (1.75) |
| Psychiatry | 4 (3.51) |
| Pulmonology | 2 (1.75) |
| Radiology | 7 (6.14) |


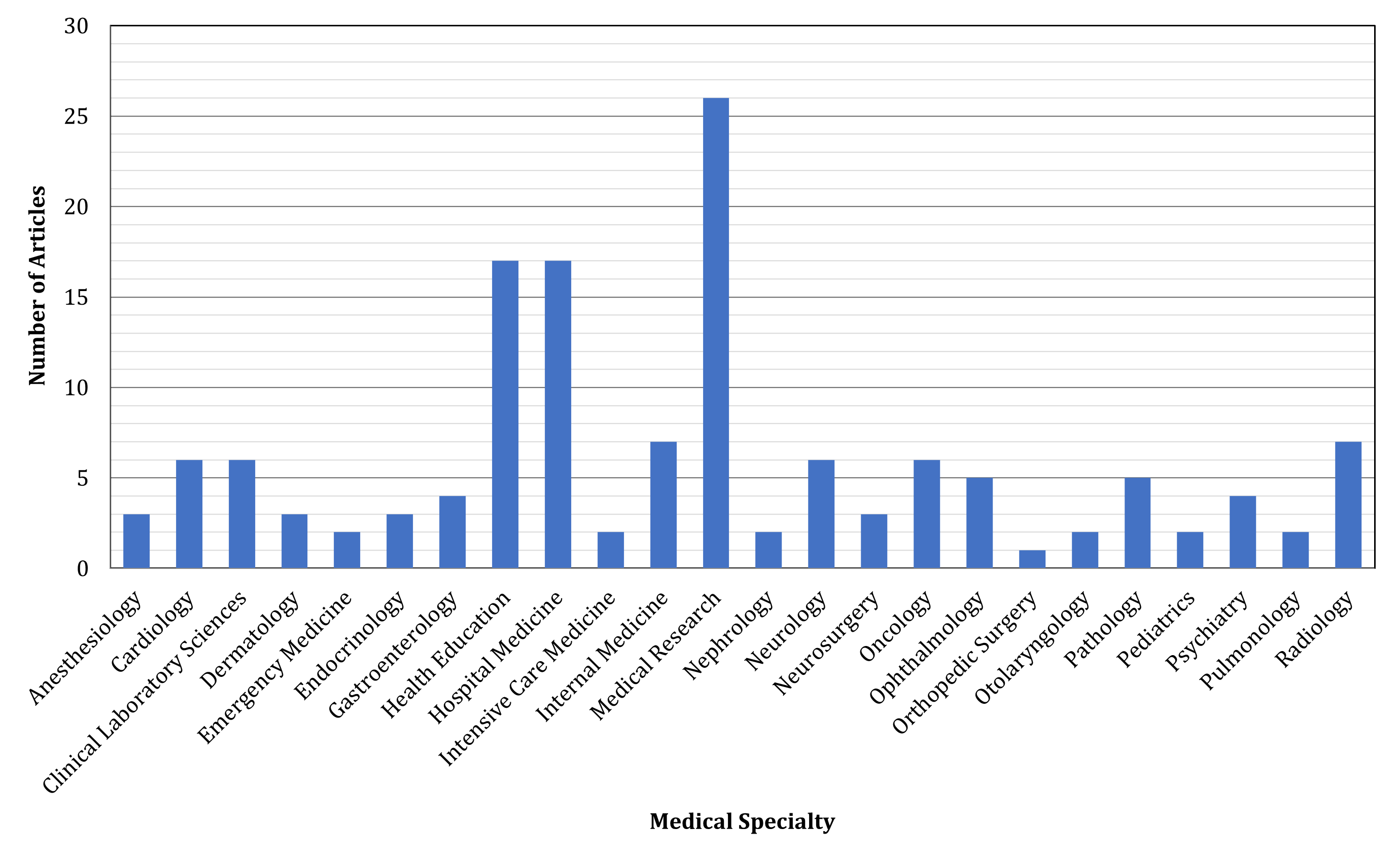


Figure S1. Medical Specialty.

### Target Use

Table S2. Target use.

| **Target Use** | **n (in %)** |
| --- | --- |
|  |  |
| (Patient) Communication | 7 (21.05) |
| Classification | 12 (16.67) |
| Decision Support | 29 (27.19) |
| Education | 13 (22.81) |
| Explanation | 28 (11.4) |
| Information Retrieval | 2 (21.05) |
| Knowledge Discovery | 6 (11.4) |
| Report Generation | 7 (3.51) |
| Summarization | 21 (6.14) |

Figure S2. Target use.

### Target Audience

Table S3. Target audience.

| **Target Audience** | **n (in %)** |
| --- | --- |
|  |  |
| Administration | 6 (5.26) |
| Lecturer | 7 (6.14) |
| Nurses | 6 (5.26) |
| Patients | 23 (20.18) |
| Physicians | 69 (60.53) |
| Researchers | 32 (28.07) |
| Students | 17 (14.91) |
| Surgeons | 5 (4.39) |

Figure S3. Target audience.

## Technical Dimensions

### Model Integration

Table S4. Model integration.

| **Model Integration** | **n (in %)** |
| --- | --- |
|  |  |
| Conceptual | 2 (1.75) |
| Usage | 65 (57.02) |
| Implementation | 27 (23.68) |
| Evaluation | 20 (17.54) |

Figure S4. Model integration.

### Model Novelty

Table S5. Model novelty.

| **Model Novelty** | **n (in %)** |
| --- | --- |
|  |  |
| Applied Existing Model | 86 (75.44) |
| Extended Existing Model | 29 (25.44) |
| Developed New Model | 3 (2.63) |
| No Model Used | 2 (1.75) |

Figure S5. Model novelty.

### Model Architecture

Table S6. Model architectures.

| **LLM Model Architecture** | **Model Family** | **n (in %)** |
| --- | --- | --- |
|  |  |  |
| Alpaca | LLaMA | 1 (0.88) |
| BARD | BARD | 9 (7.89) |
| BenTsao | LLaMA | 1 (0.88) |
| BERT | BERT | 17 (14.91) |
| Bing-Chat | BERT | 5 (4.39) |
| BioBERT | BERT | 12 (10.53) |
| BioClinicalBERT | BERT | 1 (0.88) |
| BioClinRoBERTa | BERT | 1 (0.88) |
| BioELECTRa | Other | 1 (0.88) |
| BioGPT | GPT | 3 (2.63) |
| BioLinkBERT | BERT | 1 (0.88) |
| BioMedLM | GPT | 1 (0.88) |
| BLOOM | Other | 1 (0.88) |
| BlueBERT | BERT | 1 (0.88) |
| ChatGLM | ChatGLM | 1 (0.88) |
| Claude | Other | 2 (1.75) |
| Clinical-T5 | T5 | 1 (0.88) |
| ClinicalBERT | BERT | 4 (3.51) |
| CLIP | Other | 1 (0.88) |
| Davinci | GPT | 1 (0.88) |
| DeBERTa-v3 | BERT | 1 (0.88) |
| DistilBERT | BERT | 4 (3.51) |
| DoctorGLM | ChatGLM | 1 (0.88) |
| Falcon | Other | 1 (0.88) |
| Flan-T2 | T5 | 1 (0.88) |
| Flan-T5 | T5 | 1 (0.88) |
| Flan-T5-XXL | T5 | 1 (0.88) |
| Foresight | Other | 1 (0.88) |
| GatorTron | Other | 1 (0.88) |
| GPT-2 | GPT | 4 (3.51) |
| GPT-3 | GPT | 12 (10.53) |
| GPT-3.5 | GPT | 74 (64.91) |
| GPT-4 | GPT | 41 (35.96) |
| InstructGPT | GPT | 2 (1.75) |
| LaMDA | LaMDa | 1 (0.88) |
| LLaMA | LLaMA | 10 (8.77) |
| LLaMA-2 | LLaMA | 1 (0.88) |
| LLaVA | Vicuna | 1 (0.88) |
| Longformer | BERT | 3 (2.63) |
| Med7 | Other | 1 (0.88) |
| MedBERT | BERT | 1 (0.88) |
| MiniGPT-4 | Vicuna | 2 (1.75) |
| MPT | Other | 1 (0.88) |
| not specified | not specified | 2 (1.75) |
| PaLM | LaMDa | 1 (0.88) |
| PubMedBERT | BERT | 4 (3.51) |
| PubMedGPT | GPT | 1 (0.88) |
| RadBERT | BERT | 1 (0.88) |
| REBEL | Other | 1 (0.88) |
| RoBERTa | BERT | 6 (5.26) |
| SapBERT-PubMedBERT | BERT | 1 (0.88) |
| SBERT | BERT | 1 (0.88) |
| SciBERT | BERT | 2 (1.75) |
| T5 | T5 | 2 (1.75) |
| Vicuna | Vicuna | 3 (2.63) |
| Visual-GLM | ChatGLM | 1 (0.88) |
| XLNet | Other | 2 (1.75) |

Figure S6. Model architectures.

### Data Modality

Table S7. Data modality.

| **Data Modality** | **n (in %)** |
| --- | --- |
|  |  |
| Adverse Effects | 7 (6.14) |
| Clinical Notes | 12 (10.53) |
| Diagnosis | 29 (25.44) |
| EHR | 13 (11.4) |
| Exam Questions | 28 (24.56) |
| Genomic Data | 2 (1.75) |
| Images | 6 (5.26) |
| Patient Communication | 7 (6.14) |
| Reports | 21 (18.42) |
| Symptom Description | 44 (38.6) |
| Treatment Options | 31 (27.19) |

Figure S7. Data modality.
